# Potentiometric Sensor with High Capacity Composite Composed of Ruthenium Dioxide and Poly(3,4-ethylenedioxythiophene) Polystyrene Sulfonate

**DOI:** 10.3390/ma14081891

**Published:** 2021-04-10

**Authors:** Nikola Lenar, Robert Piech, Beata Paczosa-Bator

**Affiliations:** Faculty of Materials Science and Ceramics, AGH University of Science and Technology, Mickiewicza 30, 30059 Krakow, Poland; nlenar@agh.edu.pl (N.L.); rpiech@agh.edu.pl (R.P.)

**Keywords:** high electrical capacity, stable potentiometric response, nanocomposite mediation layers, conducting polymer, poly(3,4-ethylenedioxythiophene) polystyrene sulfonate, ruthenium dioxide, potassium sensors

## Abstract

This work presents the first-time application of the ruthenium dioxide–poly(3,4-ethylenedioxythiophene) polystyrene sulfonate high-capacity composite material as a mediation layer in potassium selective electrodes, which turned out to significantly enhance the electrical and analytical parameters of the electrodes. The idea was to combine the properties of two different types of materials: a conducting polymer, poly(3,4-ethylenedioxythiophene) polystyrene sulfonate, and a metal oxide, ruthenium dioxide, in order to obtain the material for a solid-contact layer of great electrical and physicochemical parameters. The preparation method for composite material proposed in this work is fast and easy. The mediation layer material was examined using a scanning electron microscope and chronopotentiometry in order to confirm that all requirements for mediation layers materials were fulfilled. Ruthenium dioxide–poly(3,4-ethylenedioxythiophene) polystyrene sulfonate nancomposite material turned out to exhibit remarkably high electrical capacitance (of approximately 17.5 mF), which ensured great performance of designed K^+^-selective sensors. Electrodes of electrical capacity equal to 7.2 mF turned out to exhibit fast and stable (with only 0.077 mV potential change per hour) potentiometric responses in the wide range of potassium ion concentrations (10^−6^ M to 10^−1^ M). The electrical capacity of ruthenium dioxide–poly(3,4-ethylenedioxythiophene) polystyrene sulfonate-contacted electrodes characterized by electrical capacitance parameters was the highest reported so far for this type of sensor.

## 1. Introduction

Ion-selective electrodes represent nowadays a broad group of electrochemical sensors. It all started in the beginning of the 20th century when Cremer discovered the electrical properties of a thin glass membrane, which subsequently led to the design of the first potentiometric sensor by Klemensiewicz and Haber [1]. Ion-elective electrodes have significantly changed and developed over this period of time. Today, one hundred years later, we can distinguish numerous construction solutions for potentiometric sensors, which, in conjunction with the great number of functional materials known so far, creates a large potential for their applicability [2,3]. Every new material found to be appropriate for electrodes and every other design method or construction idea paves a way for more and more applications of sensors in various fields.

As to the functional materials, we usually mean the materials applied as mediation layers in solid-contact ion-selective electrodes. Mediation layers were incorporated into the construction of ion-selective electrodes after removing the internal solution, which was responsible for simplifying the ion-to-electron processes between the electronic conductor and ion-selective membrane. The elimination of the solution turned out to be beneficial for the miniaturization and flexibility of electrodes, yet disadvantageous for the transduction mechanism, which led to deterioration of the potentiometric response [3,4,5,6]. Hence, the role of the mediation layer is both to facilitate the manufacturing process of miniaturized and flexible sensors and to ease the ion-to-electron transduction [2].

When it comes to the selection of the material for the mediation layer, there are several criteria and requirements to be fulfilled and considered [7]. As mentioned, a layer should actively participate in ion-to-electron transduction without any side reactions occurring during this process [6]. As to the side reactions, the layer should also exhibit chemical stability and insensitivity to the light, atmosphere, or pH changes during the measurement [8]. The most desirable feature of materials for ISE construction seems to be high electrical capacity. The ability of the layer to store the charge determined by the double layer/redox capacitance parameter enables it to sustain the equilibrium in the presence of external disturbances and protects the electrode from the impact of the current flow during the potentiometric measurement. Electrodes with layers characterized by the high electrical capacitance parameter exhibit stable potentiometric responses over time and insensitivity to the perturbations that may occur during the measurement, e.g., power fluctuations [9].

On the other hand, the application method must ensure the proper adherence of material to electrode and at the same time not cause pollution of the ion-selective membrane by the mediation layer’s particles, which may affect the potentiometric response. Above all, materials are desired to be nontoxic, readily available, inexpensive, and easy to be applied onto electrode’s surface [2].

Finding a material that fulfills all mentioned requirements, additionally with as high electrical capacitance as possible, is a challenge for researchers focused on ion-selective electrode design and it has been a subject of studies since 1992 until present times. A great number of solid-contact functional materials have been already used as ion-to-electron transducers, including conducting polymers, carbon nanomaterials, metal and metal oxide nanomaterials, molecular redox couples, and others. Two of these groups of materials were used for the purpose of this work—to design and manufacture a solid-contact potassium-selective electrode of stable and fast potentiometric response—conducting polymer, namely poly(3,4-ethylenedioxythiophene) polystyrene sulfonate (PEDOT:PSS) and metal oxide nanomaterial, specifically ruthenium dioxide (RuO_2_). A recent trend in the design of ISEs is to combine the properties of two (or more) functional materials into one hybrid material that can be applied as a solid-contact layer. In this work, we decided to merge the conducting properties of PEDOT:PSS and high electrical capacitance of RuO_2_.

Poly(3,4-ethylenedioxythiophene) polystyrene sulfonate is an environmentally stable polymer of high conductivity yet low electrical capacitance [10], low sensitivity to interference from O_2_ and CO_2_ [9], and faster ion diffusion kinetics in comparison with other polymers [11,12]. For the first time, PEDOT:PSS was applied in ion-selective electrodes by Cadogan et al. in [13], and subsequently widely described in literature [9,14,15,16,17,18]. As reported by Bobacka in [9], PEDOT:PSS-contacted electrodes fulfill all conditions needed to consider them as robust and reliable ion sensors. The group of Lindner performed studies over PEDOT:PSS electrodes using different electrodes substrates—Au, Pt, and glassy carbon (GC)—receiving positive results in terms of the equilibration time (~10 min for applied in this research GC electrode) [14]. However, since the PEDOT:PSS polymer itself does not exhibit high electrical capacity, ion-selective electrodes with such mediation layer are also characterized by rather low (~200 μF) capacitance [9]. Hence, this work focused on enhancing polymer’s properties by the addition of high-capacity metal oxide—ruthenium dioxide—in order to obtain electrodes of reinforced stability.

Ruthenium dioxide, due to its redox properties (described in detail in our previous work [19]) and nanometric size, is characterized by high electrical capacitance and has been successfully applied in ISEs, as presented in [20,21,22].

Both materials, PEDOT:PSS and RuO_2_, have been shown to be effective as standalone materials for mediation layers in ion-selective electrodes. The aim of this work was to create a mediation layer for ISEs that combines properties of both of them into one functional material, which at the same time will ensure efficient conductivity for transduction processes and exhibit high electrical capacity.

Composite material PEDOT:PSS-ruthenium dioxide was successfully applied by Huang et al. as a supercapacitor electrode due to its highly specific capacitance [23] and by Sellam et al. in flexible pseudocapacitors thanks to its high-rate capability [24]. In the discussed nanocomposite, conducting polymer acts as a matrix for RuO_2_ particles and forms three-dimensional zones, in which ruthenium particles are loaded, creating a material with a highly active surface area.

This paper presents the first-time use of PEDOT:PSS and RuO_2_ nanocomposite as solid-contact layer in ion-selective electrodes together with the material’s preparation method, which is fast and simple as it do not require any specific apparatus or conditions.

## 2. Chemicals

Materials applied for the purpose of this work covered membrane components, mediation layer materials, and other chemicals used to prepare aqueous solutions. Membrane components included potassium ionophore I (Valinomycin), lipophilic salt-potassium tetrakis(4-chlorophenyl)borate (KTpClPB), 2-nitrophenyl octyl ether (o-NPOE). and poly(vinyl chloride) (PVC), all purchased from Sigma-Aldrich (St. Louis, MO, USA). As a solvent for membrane solution, tetrahydrofuran (THF) (Sigma Aldrich) was used.

For the preparation of mediation layers, two materials were purchased—hydrous ruthenium dioxide from Alfa Aesar (Haverhill, MA, USA) and poly(3,4-ethylenedioxythiophene) polystyrene sulfonate (PEDOT:PSS) (Sigma Aldrich in form of a 3–4% polymer in water solution). As a dispersant for solid particles of oxide and polymer, THF was used.

For the preparation of standard potassium ion solutions, the potassium chloride KCl was used. Aqueous KCl solutions for pH test were titrated with sodium hydroxide and hydrochloric acid to meet the desired pH values. All mentioned above—potassium chloride, sodium hydroxide, and hydrochloric acid—were purchased from POCH, Gliwice, Poland.

All materials were used as obtained without any further purification. For the preparation of aqueous solutions, distilled and deionized water was used.

### 2.1. Layer Hybrid Materials

**Figure 1:**
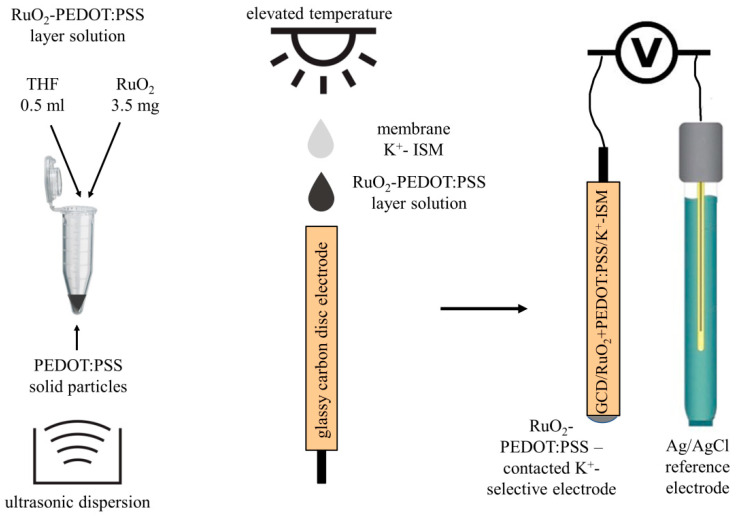
Preparation method of composite ruthenium dioxide (RuO_2_)-poly(3,4-ethylenedioxythiophene) polystyrene sulfonate (PEDOT:PSS) material and RuO_2_-PEDOT:PSS-contacted K^+^-selective electrodes.

The aim of this work was to design the new layer material that can be applied in solid-contact ion-selective electrodes. The material was created from two different components dispersed together in organic solvent: hydrous ruthenium dioxide and poly(3,4-ethylenedioxythiophene) polystyrene sulfonate (PEDOT:PSS). The method of material preparation was fast and simple and did not require any specific apparatus, as it is presented in the Figure 1. To obtain the layer material, 0.5 mL of PEDOT:PSS water solution was heated to remove the water by the evaporation process. Solid particles of polymer (~17 mg) were mixed with 3.5 mg of ruthenium dioxide and together, materials were dispersed ultrasonically (5 h) in 0.5 mL of THF. The prepared material solution was applied onto electrodes, which were studied in the scope of this work.

### 2.2. Electrodes Preparation

For the purpose of this work—to evaluate the influence of hybrid RuO_2_-PEDOT:PSS material on ISE properties—two groups of polymer membrane-based electrodes were prepared: one group of solid-contact electrodes and one group of coated-disc electrodes. The schematic preparation method of RuO_2_-PEDOT:PSS-contacted electrodes is presented in Figure 1. The preparation procedure was as follows. Glassy carbon (GC) disc electrodes were polished on alumina slurries and rinsed with water and methanol. Layers were dropped onto a preheated surface of electrodes (3 min, 80 °C) in the form of a solution (20 μL) using drop casting method. A THF-based layer was dried at room temperature until the solvent evaporated. Onto the dry RuO_2_ + PEDOT:PSS layers, ion-selective membrane was casted using also the drop casting method. The potassium-selective membrane composition was as presented: ionophore I 1.10% (*w*/*w*), KTpClPB 0.25% (*w*/*w*), o-NPOE 65.65% (*w*/*w*), PVC 33.00% (*w*/*w*). All membrane components of total weight 0.25 g were dissolved in 2 mL of THF. Using this procedure, 3 items of GC/RuO_2_ + PEDOT:PSS/K^+^-ISM electrodes were prepared. As a control—to evaluate the influence of designed layers—a group of coated-disc electrodes (GCD/K^+^-ISM) was obtained by dropping the membrane solution directly onto the electrodes’ disc surface.

All prepared electrodes were conditioned in 0.01 M KCl solution prior to every measurement.

### 2.3. Conducted Measurements

In the scope of presented work both composite material for solid-contact layer and solid-contact ion-selective electrodes were examined using various methods. The examination of layers was done by scanning electron microscopy and chronopotentiometry method. Ion-selective electrodes were also tested using the chronopotentiometry method in order to determine their electrical properties and by the potentiometry method to evaluate their analytical parameters.

Scanning electron microscopy (LEO 1530, Carl Zeiss, Jena, Germany) was used for the examination of layer’s microstructure.

A chronopotentiometry technique was used for the layer and electrode examination. Using this electrochemical method and equations presented by Bobacka in [9] it is possible to estimate such electrical parameters as electrical capacitance, resistance, and potential drift of both layers and ready-to-use electrodes with an ion-selective membrane. For this purpose, the Autolab (Eco Chemie AUT32N.FRA2-AUTOLAB, ΩMetrohm, Herisau, Switzerland) analyzer with NOVA software [*Nova*, version 2.1; Software for analysis of experimental data and simulation; ΩMetrohm: Herisau, Switzerland, 2016] was used. Firstly, only layers deposited onto GC electrodes and subsequently layers covered with membranes were tested in a 3-electrode cell composed of a working electrode (glassy carbon electrode with studied layer and optionally ion-selective membrane), a reference electrode (Ag/AgCl electrode type 6.0733.100, ΩMetrohm), and an auxiliary electrode (glassy carbon rod). All tests were carried out in 0.01 M KCl solution.

The potentiometry method was applied to determine the analytical parameters of the studied solid contact electrodes with a RuO_2_ + PEDOT:PSS layer and to compare obtained results with those of the coated-disc electrode. The 16-channel Lawson Labs potentiometer was used to detect the changes in the electromotive force (EMF) of the cell. Designed ion-selective electrodes were studied versus reference electrodes (Ag/AgCl electrode) and in the presence of auxiliary electrodes (platinum wire). For the potentiometry method, standard KCl solutions from 10^−7^ to 10^−1^ M were used.

## 3. Results

### 3.1. SEM

**Figure 2:**
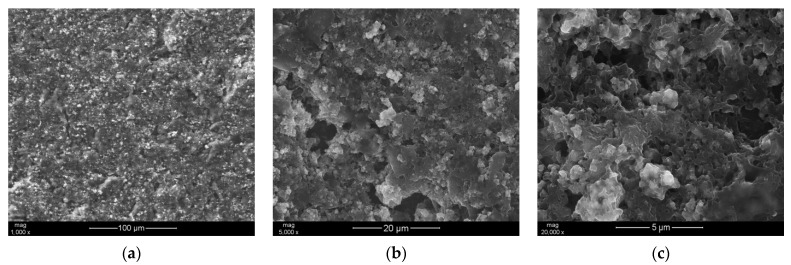
SEM images showing the morphology of the RuO_2_—PEDOT:PSS composite. (**a**–**c**) Magnification series from the lowest to the highest magnification.

The examination of RuO_2_—PEDOT:PSS composite material began with analyzing the microstructure of material using scanning electron microscopy (SEM). Designed material was casted onto aluminum pads and, after drying, scanned with a beam of electrons in the sample chamber of the electron microscope. Figure 2 presents SEM magnification series for examined nanocomposite (from the lowest magnification equal to 1000×—Figure 2a, to the highest of 20,000×—Figure 2c). Scans depict the presence of ruthenium dioxide nanometric particles (lighter spots) spread out evenly on the flake layers of the PEDOT:PSS polymer.

When comparing all obtained SEM images, it can be seen that the microstructure of the designed nanocomposite is rough and uneven. This roughness of RuO_2_ + PEDOT:PSS layer indicates high surface area of the material, that in further analysis should translate into high values of the electrical capacitance parameter.

### 3.2. Chronopotentiometry

**Figure 3:**
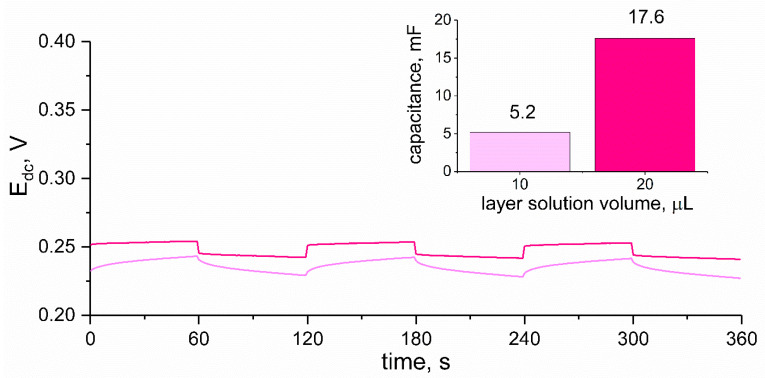
Chronopotentiograms recorded for RuO_2_-PEDOT:PSS material dispersed in tetrahydrofuran (THF). Light color corresponds to the lower thickness of the solid-contact layer (10 μL) and dark color indicates greater thickness (20 μL). Inset: electrical capacitance values compared for studied thicknesses of solid-contact layer.

When designing solid-contact ion-selective electrodes, the first aspect to consider is the choice of material for the solid-contact layer, and further the choice of dispersant (solvent) and the optimal thickness of the layer. For this purpose, the chronopotentiometry method turned out to be useful, as this method allowed us to determine the value of the electrical capacitance parameter of the mediation layer. As mentioned in the introduction section, the higher the electrical capacitance value, the better, therefore by comparing the parameter values obtained for different versions of layers, it was possible to select the best type of mediation layer and its most favorable thickness. As dispersant for the composite layer, tetrahydrofuran was considered to be the most suitable, as this solvent was successfully used in our previous work for the preparation of similar materials (POT (Poly(3-octylthiophene-2,5-diyl)-RuO_2_ composite). As to the thickness of the layer, it was measured by the amount of the material solution dropped onto the electrode’s surface in the process of electrode preparation (by drop casting method). Three layer thicknesses were considered: after applying 10 μL, 20 μL, and 30 μL of RuO_2_-PEDOT:PSS solution in THF. Layers (of appropriate amount) were cast onto the GC disc electrodes’ surfaces and, after drying, one-by-one each electrode was connected to the Autolab analyzer in the three-electrode cell together with reference and auxiliary electrodes. The three electrodes were analyzed in 10^−2^ M K^+^ ion standard solution and the potential was recorded with the current flow set on 1 μA. After every minute the sign of current changed (starting with positive sign and ending the measurement with negative). All six steps were registered through the time of 360 s and the obtained chronopotentiograms are presented in the Figure 3. Inset to the Figure 3 presents electrical capacitance (C) values calculated using the ΔE_dc_/Δt = I/C equation based on the recorded potential (E_dc_) while a current (I) of 1 μA was applied to the electrode in the time (t) of 360 s.

For electrodes covered with 30 μL of RuO_2_-PEDOT:PSS solution, it was not possible to obtain a stable chronopotentiogram since the particles of composite materials were detached from electrode’s surface during the measurement. This amount of solution turned out to create layer that was too thick and the adherence was not sufficient. For the other two examined amounts, it was possible to determine the capacitance of layers based on the obtained chronopotentiograms, which was greater for 20 μL (dark line color) than for 10 μL (light line color) of the THF-based solution.

Based on the obtained electrical capacitance values (6.2 mF for smaller layer thickness and 17.6 mF for greater thickness), the selected amount of solution was set as 20 μL. Further measurements were conducted using electrodes with 20 μL of RuO_2_-PEDOT:PSS THF-based solution as a mediation layer.

**Figure 4:**
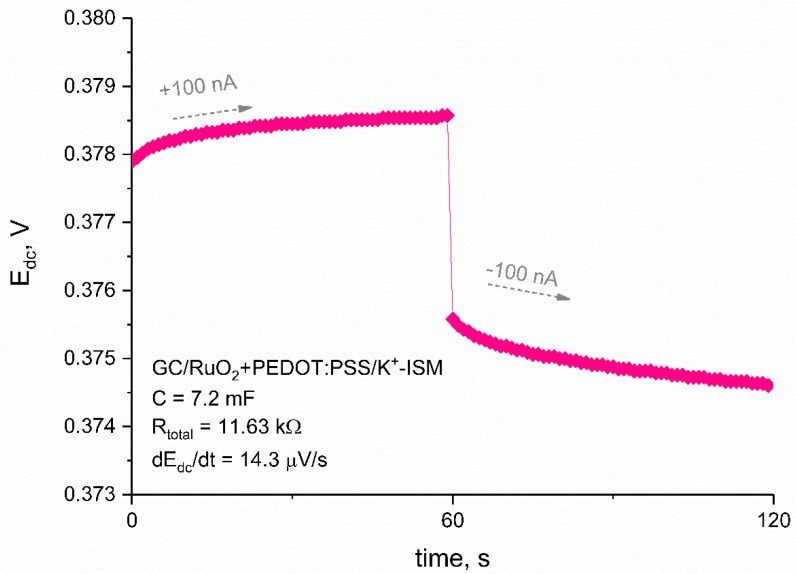
First two steps of chronopotentiograms recorded for glassy carbon (GC)/RuO_2_ + PEDOT:PSS/K^+^-ISM electrode with electrical parameter values.

After the layer examination, solid-contact ion-selective electrodes with potassium selective membrane were prepared using the selected amount (20 μL) of the mediation layer. Ready-to-use electrodes were also tested using the chronopotentiometry method. The measurement was conducted in the same conditions as it was for the layer itself, yet the applied current was lower and equaled 100 nA. Figure 4 presents the two first steps of the recorded chronopotentiograms—0–60 s recorded for +100 nA current and 60–120 s for −100 nA current.

Electrical capacitance values for studied electrodes were calculated for the linear parts of the chronopotentiograms and the obtained value equaled to 7.2 ± 1.3 mF for GC/RuO_2_ + PEDOT:PSS/K^+^-ISM electrode.

Using the chronopotentiometry method, two other electrical parameters can be calculated: resistance and potential drift of electrodes. The total resistance of the electrodes was calculated using the value of potential jump ΔE_dc_ and current I: R_total_ = ΔE_dc_/2I and equaled 15.01 ± 0.08 kΩ for GC/RuO_2_ + PEDOT:PSS/K^+^-ISM electrode. The potential drift in the forced current conditions (ΔE_dc_/Δt) equaled 14.3 ± 2.5 μV/s for GC/RuO_2_ + PEDOT:PSS/K^+^-ISM electrode, respectively.

What should be empathized here is that the designed GC/RuO_2_ + PEDOT:PSS/K^+^-ISM electrode exhibited remarkable electrical parameters in comparison with results presented so far in the literature for K^+^-selective electrodes. For PEDOT:PSS-contacted electrodes, the electrical capacity reported by Bobacka in [9] equaled 0.2 mF, while for poly(3,4-ethylenedioxythiophene) doped with chloride—0.30 mF. The addition of PEDOT:PSS polymer to ruthenium dioxide allowed for the sevenfold increase of the capacitance value, as for the ruthenium dioxide-based electrodes, the maximal obtained capacitance value equaled 1.07 mF [19]. Electrical capacity of designed RuO_2_ + PEDOT:PSS-contacted electrodes turned out to be significantly higher than the one of RuO_2_ + POT-contacted K^+^-selective electrodes (1.17 mF for the most optimal parameters of layer [21]). The comparison of electrical capacitance and potential stability (characterized by potential drift value) of RuO_2_ + PEDOT:PSS-contacted electrode with other K^+^-selective electrodes reported so far in literature is presented in the Table 1.

This comparison proves that poly(3,4-ethylenedioxythiophene):poly(styrene sulfonate) is an unquestionably noteworthy material to be considered as one of the components of composite mediation layers. In fact, the electrical capacitance value characterizing the RuO_2_ + PEDOT:PSS nanocomposite—based ion-selective electrodes­—is, to the best of our knowledge, the highest value reported for ion-selective electrodes with PCV membrane [2].

### 3.3. Potentiometry

#### 3.3.1. Potentiometric Response

Properties of mediation layers decide on electrical parameters of ion-selective electrodes and further define their analytical performance. Analytical parameters of designed ion-selective electrodes were examined with the use of the potentiometry method. Firstly, electrodes were characterized by indicating the slope of the calibration curve and the standard potential during calibration process. The calibration curve was recorded as a function of electromotive force (EMF) vs. logarithm of potassium ion activity. The potentiometric response of GC/RuO_2_ + PEDOT:PSS/K^+^-ISM electrodes was tested in KCl standard solution with K^+^ ion concentration from 10^−7^ to 10^−1^ M. Solid-contact ion-selective electrodes were examined in the presence of coated-disc electrodes to evaluate the influence of composite material presence on the potentiometric response.

**Figure 5:**
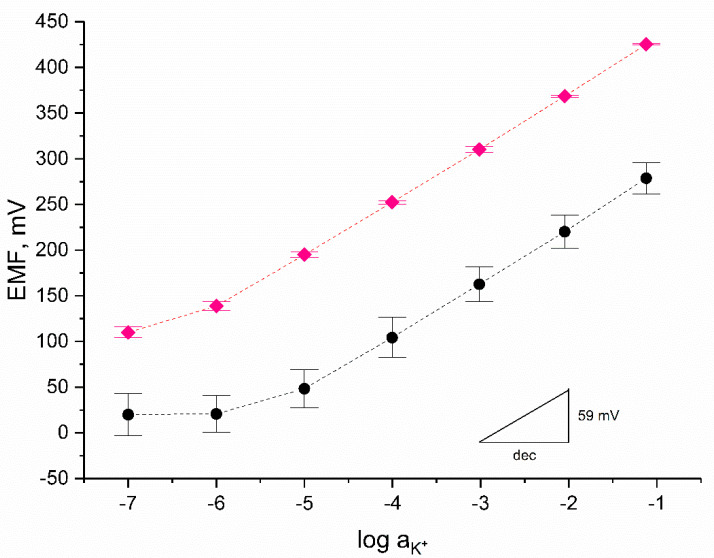
Calibration curves prepared on the basis of average electromotive force (EMF) values of GC/RuO_2_ + PEDOT:PSS(THF)/K^+^-ISM (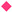) and GCD/K+-ISM (●) electrodes measured after 24, 48, and 72 h of conditioning (*n* = 3) with error bars depicting repeatability of potentiometric response.

Electrodes were tested using the same procedure through the time of three days, which allowed us to determine their repeatability. Figure 5 presents the exemplary potentiometric response of all three tested groups of electrodes recorded after 24, 48, and 72 h of electrode conditioning in 10^−2^ M K^+^ ion solution. Repeatability of electrodes’ potential responses is given by the standard deviation value in the form of error bars. Solid-contact ISEs with RuO_2_-PEDOT:PSS composite material as the solid-contact layer exhibited great repeatability over three days of measurement. For comparison, in the case of the GCD/K^+^-ISM electrode, the standard deviation values of the measured potentials were of much higher values which proves that the presence of the mediation layer positively influences the repeatability of ion-selective electrodes.

Analytical parameters (slope of calibration curve, standard potential, and linear range) of RuO_2_-PEDOT:PSS-based electrodes were collected in Table 2. The average values presented in the table were calculated based on the results obtained for three items in each group of electrodes. Taking into consideration the standard deviation values calculated for each group, it is possible to determine the convergence of potentiometric responses of electrodes representing one group. PEDOT:PSS-based electrodes exhibited great reproducibility of potentiometric response with standard deviation of only 0.5 mV for calibration curve slope and a few mV for standard potential.

For both groups of RuO_2_-PEDOT:PSS-based electrodes, the near-Nernstian response was observed for K^+^ ions with a concentration between 10^−6^ and 10^−1^ M. In contrast, for coated-disc electrodes, the linear response was observed in narrower range—from 10^−5^ to 10^−1^ M. It can be therefore concluded that the presence of a composite material mediation layer beneficially affects the potentiometric response of electrodes in terms of the linear range.

#### 3.3.2. Potential Stability

**Figure 6:**
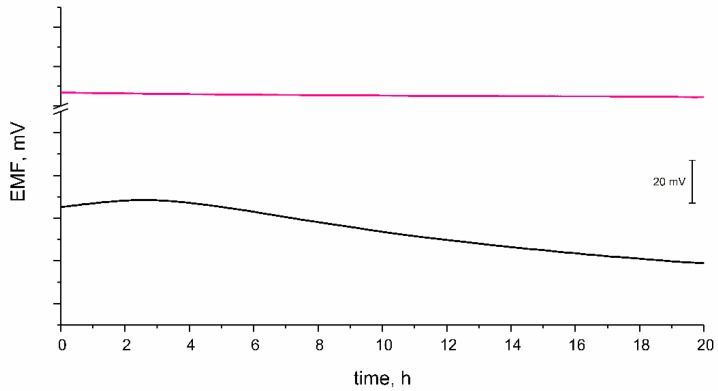
Stability of potential presented as potential change in time for one electrode representing each tested group. GC/RuO_2_ + PEDOT:PSS(THF)/K^+^-ISM (pink line), GC/K^+^-ISM (black line).

One of the challenges that researchers focused on ion-selective electrodes are facing is designing the sensor with the potential response perfectly stable over the time of measurement. The stability of potentiometric response was tested during a 21-h long measurement, which is presented in the Figure 6. The potential was recorded while electrodes were placed in 10^−2^ M K^+^ ion standard solution. Based on the obtained results, the potential drift was calculated. For the GC/RuO_2_ + PEDOT:PSS/K^+^-ISM electrode, the potential change equaled to 0.077 mV/h. In contrast, for the coated-disc electrode without composite material layer the potential drift was much more significant and equaled to 1.76 mV/h. It can be concluded that the presence of the mediation layer consisting of ruthenium dioxide and poly(3,4-ethylenedioxythiophene) is favorable for ion-selective electrodes in the terms of the potential stability. According to the data presented by Bobacka in [9], the potential drift of a single component PEDOT:PSS-contacted electrode is significantly higher (of approximately 0.18 mV/h) than the of designed nanocomposite RuO_2_ + PEDOT:PSS-contacted electrode.

#### 3.3.3. Light Sensitivity Test

**Figure 7:**
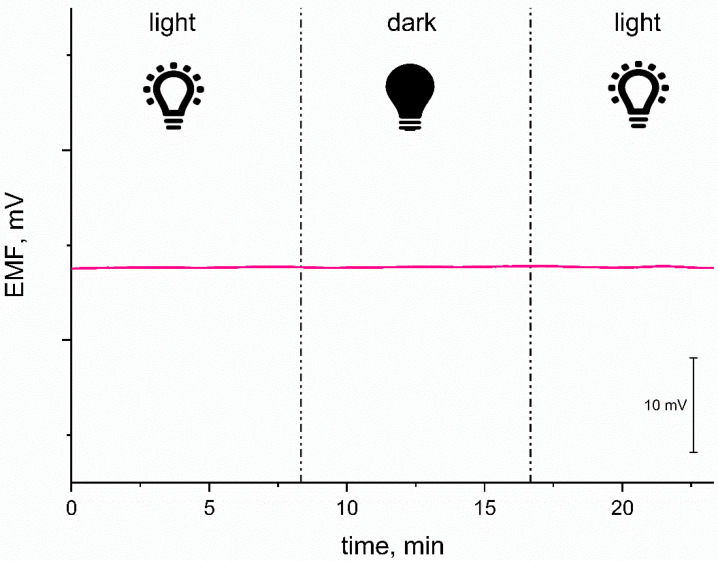
Potentiometric response of studied GC/RuO_2_ + PEDOT:PSS/K^+^-ISM electrode (one electrode representing the group of solid-contact electrodes) showing its insensitivity to the light exposition.

Regardless of the presence of the conducting polymer in the composite material used as a mediation layer, which tends to be susceptible to the light exposure, the light sensitivity test was conducted for designed solid-contact electrodes. The test was performed in the 10^−2^ M K^+^ ions standard solution and EMF was monitored while changing the light intensity—from very bright room light to complete dark conditions, followed again by light conditions. As presented in Figure 7, the potential response of the tested exemplary RuO_2_-PEDOT:PSS-contacted electrode was stable during experiment and there was no reaction to the light exposition. The test confirmed that the electrodes presented in this work are not susceptible to light exposure.

#### 3.3.4. pH Sensitivity Test

On the other hand, regardless of the presence of ruthenium dioxide in the composite material, which exhibits pH dependence, the pH sensitivity test was conducted for designed solid-contact electrodes.

**Figure 8:**
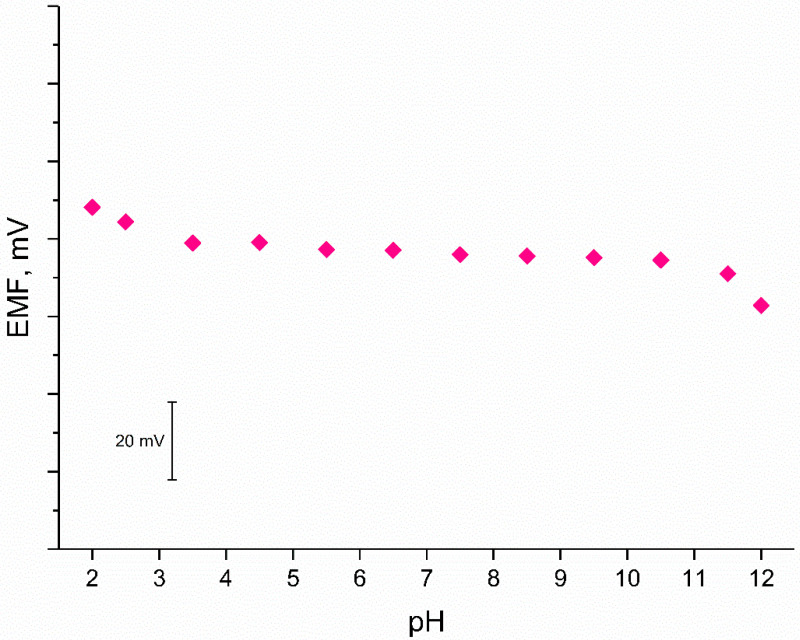
Potentiometric response of studied GC/RuO_2_ + PEDOT:PSS/K^+^-ISM electrode (one electrode representing the group of solid-contact electrodes) showing its insensitivity to a changing pH value.

The test was conducted in the solutions of constant potassium ion concentration and changing pH value (from 2 to 12). Standard 10^−2^ M K^+^ ion solution was titrated with sodium hydroxide to obtain solutions of pH scope from 6–12 and hydrochloric acid was used to obtain lower pH values. As can be seen in Figure 8, stable EMF value was observed for solutions of pH from 3.5 to 10. The test has shown that the electrode presented in this work is not susceptible to the changes in the pH values between 3.5 and 10.

#### 3.3.5. Water Layer Test

Ion-selective electrodes, after being transformed from conventional electrodes with internal solution into all-solid-state electrodes, with membranes placed directly onto the electronic conductor, were characterized by the issue of water layer formation. After being repeatedly placed into water solutions during a series of measurements and conditioning, all-solid-state electrodes tended to absorb water and the so called water layer was formed in the interface between ion-selective membrane and the electrode’s surface. The presence of this thin water film led to the potential drift of potentiometric response and may be the cause of shortening the lifetime of electrodes by deteriorating the adherence of the membrane to the electrode. Taking this into consideration, the possibility of formation of such a layer was evaluated during the water layer test.

The water layer test was conducted according to procedure presented by Fibbioli et al. in [25] and Guzinski et al. in [26]. Designed RuO_2_-PEDOT:PSS-contacted electrodes were placed into 10^−2^ M K^+^ ion standard solution for 20 h, then into 10^−2^ M Na^+^ ion standard solution for several hours, and eventually placed back into the potassium ion solution for a longer period of time. During the test the EMF was recorded with time to observe the possible potential drift when changing the primary ion in the analyzed solution.

**Figure 9:**
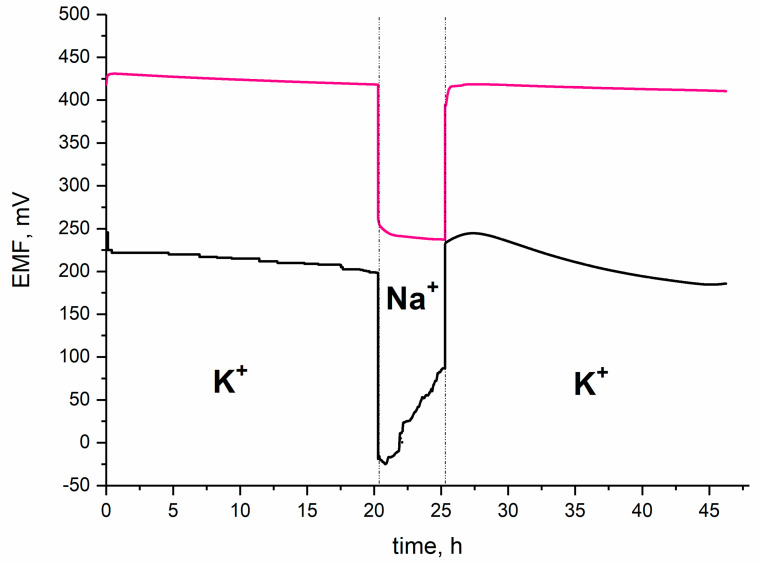
Water layer test conducted for GC/RuO_2_ + PEDOT:PSS/K^+^-ISM and GC/K^+^-ISM electrode (for one electrode representing each tested group). GC/RuO_2_ + PEDOT:PSS(THF)/K^+^-ISM (pink line), GC/K^+^-ISM (black line).

As presented in the Figure 9, for GC/RuO_2_ + PEDOT:PSS/K^+^ electrode the potential drift was not observed when changing the KCl into NaCl solution and the potentiometric response was fast and stable. In contrast, coated-disc electrodes exhibited substantial drift of EMF, indicating the presence of a water layer formed under the ion-selective membrane.

## 4. Conclusions

K^+^-selective solid-contact electrodes presented in this work exhibit great electrical and analytical parameters. A composite material formed using two different types of material, conducting polymer (PEDOT:PSS) and metal oxide (RuO_2_), fulfills all the requirements for a mediation layer in ion-selective electrodes. The designed material turned out to exhibit remarkable electrical capacity, evaluated with chronopotentiometry method (with an electrical capacitance parameter equal to 17.5 mF), which resulted in high electrical capacitance of RuO_2_-PEDOT:PSS electrodes (of approximately 7 mF). This electrical capacitance value is, to the best of our knowledge, the highest value reported for ion-selective electrodes with PCV membrane.

Consequently, thanks to the great electrical parameters of electrodes, the potentiometric response was fast and stable (with potential drift equal to 0.077 mV/h). RuO_2_-PEDOT:PSS material ensured the lack of a water layer, proven by the stable potentiometric response during the water layer test.

The tested group of GC/RuO_2_ + PEDOT:PSS/K^+^-ISM electrodes were characterized by near-Nernstian responses in the potassium ion concentrations from 10^−6^ M to 10^−1^ M. In comparison with coated-disc electrodes, RuO_2_-PEDOT:PSS-contacted electrodes exhibited more stable potentiometric responses in wider K^+^-ions concentration, which confirms beneficial impact of the mediation layer on the sensor’s properties.

Even though PEDOT:PSS tends to be susceptible to changing light conditions and ruthenium dioxide exhibits responsivity to pH changes, electrodes with composite material built of those two components showed insensitivity to both light exposition and changing pH value. Designed RuO_2_-PEDOT:PSS nanocomposite-based electrodes are insensitive to unstable conditions that may occur during measurement, which makes them the reliable tool for potassium ion determination.

The method proposed for both composite material and electrodes preparation is fast and simple and does not require the use of specific equipment or conditions.

## Figures and Tables

**Table 1:** Electrical and potentiometric parameters compared for a group of coated-disc electrodes (GCD)/ solid-contact (SC)/K^+^-ISM electrodes with various materials applied as solid-contact (SC) layers.

Electrode	Capacitance Value (μF)	Potential Drift (μV/s)	Reference
GCD/PEDOT(CNT)/K^+^-ISM	83	12	[18]
GCD/RuO_2__⋅_xH_2_O/K^+^-ISM	1233	81	[19]
GCD/PEDOT:PSS/K^+^-ISM	204	4.9	[9]
GCD/PEDOT(Cl)/K^+^-ISM	300	3.3	[9]
GCD/RuO_2_ + POT/K^+^-ISM	1167	86	[21]
GCD/POT/K^+^-ISM	1.25	798	[21]
GC/RuO_2_ + PEDOT:PSS/K^+^-ISM	7200	14.3	this work

**Table 2:** Potentiometric response towards K^+^ ions for the group of RuO_2_-PEDOT:PSS-based electrodes and coated-disc electrodes (*n* = 3 items), with standard deviation values depicting reproducibility of electrodes’ responses within a group.

Group of Electrodes	Slope (mV/dec)	Standard Potential (mV)	Linear Range (M)
GC/RuO_2_ + PEDOT:PSS/K^+^-ISM	58.93 ± 0.49	382 ± 3	10^−1^–10^−6^
GC/K^+^-ISM	59.31 ± 0.61	343 ± 15	10^−1^–10^−5^

## Data Availability

Data available in a publicly accessible repository.
